# Open Data in Global Environmental Research: The Belmont Forum’s Open Data Survey

**DOI:** 10.1371/journal.pone.0146695

**Published:** 2016-01-15

**Authors:** Birgit Schmidt, Birgit Gemeinholzer, Andrew Treloar

**Affiliations:** 1 State and University Library, University of Göttingen, Göttingen, Germany; 2 Systematic Botany, University of Giessen, Giessen, Germany; 3 Australian National Data Service, Melbourne, Australia; University of Colorado, UNITED STATES

## Abstract

This paper presents the findings of the Belmont Forum’s survey on Open Data which targeted the global environmental research and data infrastructure community. It highlights users’ perceptions of the term “open data”, expectations of infrastructure functionalities, and barriers and enablers for the sharing of data. A wide range of good practice examples was pointed out by the respondents which demonstrates a substantial uptake of data sharing through e-infrastructures and a further need for enhancement and consolidation. Among all policy responses, funder policies seem to be the most important motivator. This supports the conclusion that stronger mandates will strengthen the case for data sharing.

## Introduction

The Belmont Forum, a group of high-level representatives from major funding agencies across the globe, coordinates funding for collaborative research actions to address the challenges and opportunities of global environmental change. To collaboratively develop capable e-infrastructures to meet the data arising from the Belmont Challenge and the Future Earth agenda, a multi-phased E-Infrastructure and Data Management Collaborative Research Action (CRA) was initiated in late 2013. This was given the task to develop a strategy and implementation plan to further shape strategic science policies, outlining what can be done better, in a multilateral way, to fund and support global environmental change research. Note that this CRA was only focussed on defining possible actions in support of this agenda—the Belmont Forum will need to decide which actions it is able to support.

A survey, developed by the Belmont Forum working group on open data (one of 6 working groups under this CRA) invited researchers of various science communities, interested laypersons, government employees, and others who are providing and/or using open data in the scope of global environmental change, or are planning/interested in doing so in the future, to share their views and experiences on data publishing, access and (re)use. The main aim was to learn more about:

- Key open data activities in various communities dealing with global environmental change to identify leading examples of best practice from a user perspective;
- Areas where users’ desire to share could be enhanced by new/other developments;
- Barriers to “open data sharing” from a user perspective (as either a data provider or data user).

## Methods

From 16 September to 12 November 2014, 1330 responses to the Belmont Forum’s Open Data Survey were collected through a web survey (made available using SurveyMonkey, cf. S1 Appendix). Dissemination channels for invitations to complete the survey included about 20 disciplinary and professional mailing lists targeting researchers, data scientists, data managers and technologists from the environmental sciences, earth sciences, marine and polar sciences, biodiversity, as well as social and economic sciences. On 25 September 2014 the survey was distributed to all authors of the open access publisher Copernicus Publications (c 29,000 recipients with a strong focus on geo and space sciences) which generated a peak in responses of over 750 responses, dropping down within a few days after the mailing (539 on 25 Sept., 161 on 26 Sept., 31 and 30 responses on 27 and 28 Sept.). A potential bias of the respondents should be taken into account as these authors are likely better informed about the topic of the survey than the general author population. In addition, as the survey was known to be forwarded on by recipients to other mailing lists to which they belonged, no assumptions can be made about the underlying base population for the survey. This needs to be borne in mind when considering the degree to which the responses are representative of a population as a whole.

All of the 19 questions of the survey were non-mandatory, i.e. could be skipped by respondents. Some visitors of the survey left at early stages of the questionnaire, therefore we have only used those 1253 responses which got as far as, and provided information about, their employment role.

For the analysis the statistics software R and in particular the Likert package [1] were used. For the publication of the survey data [2] free text answers have either been lightly edited (question 4, other), or are provided in separate files (questions 8, 15, 16, 17).

### Ethics statement

As the Belmont Forum’s international working group on Open Data is not directly affiliated to a specific institution but works across a number of institutions (and indeed countries) the survey was not checked by an institutional Ethics Committee. The corresponding author’s institution, the University of Göttingen, does not have an ethics committee in place for all research areas; a committee is currently only under development. However, during the development of the survey an expert from the social sciences was consulted on the design and the collection of individual-related data. To secure privacy all data were collected via a web survey (SurveyMonkey) and analyzed anonymously. In particular, aggregation was already built into the questionnaire (age groups, employment categories, disciplinary areas, no gender category, no details about academic status, etc.) and all IP addresses were removed after the data collection. All free text data was aggregated and analysed separately. All participants were informed that the survey was anonymous and voluntary, and that the purpose was to analyse the views of disciplinary communities regarding the provision/use of open data. Participants were informed that the study results were to be published.

## Results

**Fig 1:**
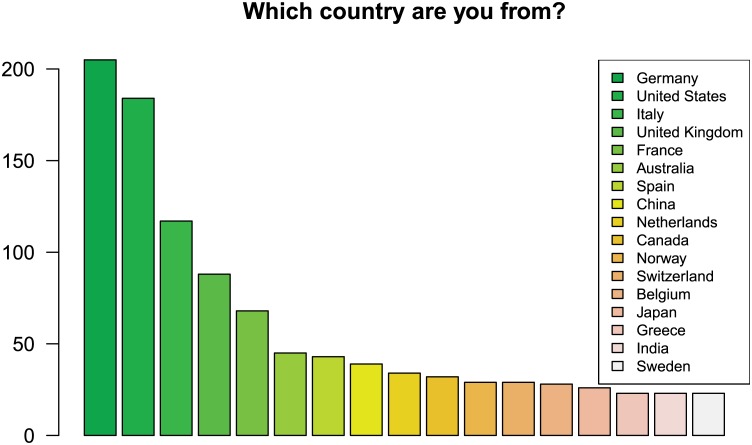
Countries with more than 20 responses. The majority of responses came from central Europe and the United States.

Overall, data was collected from 80 countries, based on information provided by 1248 respondents (cf. Table 1). Fig 1 displays the 17 countries with more than 20 responses. The “other countries” category contains 212 responses and is not shown.

**Table 1:** Countries.

Country	Frequency	Percentage
Germany	205	16.4
United States	184	14.7
Italy	117	9.4
United Kingdom	88	7.1
France	68	5.4
Australia	45	3.6
Spain	43	3.4
China	39	3.1
Netherlands	34	2.7
Canada	32	2.6
Norway	29	2.3
Switzerland	29	2.3
Belgium	28	2.2
Japan	26	2.1
Greece	23	1.8
India	23	1.8
Sweden	23	1.8
Austria	18	1.4
Finland	15	1.2
Russia	14	1.1
Brazil	11	0.9
Portugal	10	0.8
Other (less than 10 resp.)	144	11.5

Table shows frequencies and valid percentages for each country (n = 1248).

Information about age was provided by 1247 individuals (cf. Table 2). The average age of respondents was about 43 years (based on the average age in each group, and of 20 and 90 years for the extremes). The median is represented by the age group of 41 to 45 years. Information about age was not supplied by six individuals.

**Table 2:** Age groups.

Age	Frequency	Percentage
< = 20	1	0.1
21–25	22	1.8
26–30	142	11.4
31–35	232	18.6
36–40	208	16.7
41–45	168	13.5
46–50	152	12.2
51–55	116	9.3
56–60	101	8.1
61–65	53	4.3
66–70	32	2.6
71–75	9	0.7
76–80	7	0.6
81–85	3	0.2
>90	1	0.1

Table shows frequencies and valid percentages for each age category (n = 1247).

Of all respondents who provided information about their employment role (n = 1,248, cf. Table 3), 70.1% (878 responses) percent work in academic positions, 17.9% (224 resp.) in government, 5.6% (70 resp.) in non-profit institutions, 2.6% (32 resp.) in business and 0.3% (4 resp.) in media institutions. In addition, 3.6% (45 resp.) saw themselves in other roles, e.g. in other types of research institutions, international organizations, or are independent researcher/consultant or retired researchers. Some belonged to the first five categories but may have a double role.

**Table 3:** Employment role.

Employment	Frequency	Percentage
Academia	878	70.1
Government	224	17.9
Non profit	70	5.6
Other	45	3.6
Business	32	2.6
Media	4	0.3

Table shows frequencies and valid percentages for each employment category (n = 1253).

Overall, of all respondents that provided information about their data professional role (n = 1248), 82.3% (1025 respondents) saw themselves as data users, 57.6% as data providers (718 respondents), and 25.3% as data managers (315 respondents) (multiple answers were allowed). About 5.3% (66 respondents) of all respondents saw themselves in other or multiple data roles, and/or were unsure on how to classify themselves, e.g. as researchers, (data) librarian, software developer, administrator etc.

Respondents were allowed to select more than one disciplinary area. Of those who provided information (n = 1232, cf. Table 4) the majority of respondents belonged to earth and environmental sciences (68.7%, 846 responses) and climate and atmospheric sciences (31.3%, 386 responses). In addition, there were at least 50 responses from the biological sciences (20.9%, 258 responses), physical sciences (13.1%, 162 responses), engineering (7.1%, 88 responses), computer science (6.9%, 85 responses), social sciences (5.4%, 66 responses), agricultural and veterinary sciences (4.3%, 53 responses) and the chemical sciences (4.1%, 50 responses).

**Table 4:** Subject disciplines.

Discipline	Frequency	Percentage
Earth sciences and environmental sciences	846	68.7
Climate and atmospheric sciences	386	31.3
Biological sciences	258	20.9
Physical sciences	162	13.1
Engineering	88	7.1
Computer sciences	85	6.9
Social sciences	66	5.4
Agricultural and veterinary sciences	53	4.3
Chemical sciences	50	4.1
Other discipline	40	3.2
Health sciences	22	1.8
Economics	21	1.7

Table shows frequencies and valid percentages for each disciplinary category, multiple answers were allowed (n = 1232).

Some disciplines feature a substantial overlap, e.g. every second physicist who responded to the survey also belonged to climate and atmospheric sciences (86 of 162 responses, 53.1%, cf. Table 5).

**Table 5:** Overlap of disciplines in global environmental change research.

	Phys	Chem	Earth	Bio	Agric.	Social	Comp	Clim	Health	Engin	Econ
Physic. sc.	162	23	115	17	6	6	17	86	4	22	3
percentage	100.0	14.2	71.0	10.5	3.7	3.7	10.5	53.1	2.5	13.6	1.9
Chem. sc.	23	50	40	11	4	4	8	22	1	3	4
percentage	46.0	100.0	80.0	22.0	8.0	8.0	16.0	44.0	2.0	6.0	8.0
Earth sc.	115	40	846	114	32	36	53	249	11	64	14
percentage	13.6	4.7	100.0	13.5	3.8	4.3	6.3	29.4	1.3	7.6	1.7
Biol. sc.	17	11	114	258	20	12	25	29	9	9	5
percentage	6.6	4.3	44.2	100.0	7.8	4.7	9.7	11.2	3.5	3.5	1.9
Agric. sc.	6	4	32	20	53	6	3	14	2	5	6
percentage	11.3	7.5	60.4	37.7	100.0	11.3	5.7	26.4	3.8	9.4	11.3
Social sc.	6	4	36	12	6	66	12	21	7	7	10
percentage	9.1	6.1	54.5	18.2	9.1	100.0	18.2	31.8	10.6	10.6	15.2
Comp. sc.	17	8	53	25	3	12	85	23	5	15	5
percentage	20.0	9.4	62.4	29.4	3.5	14.1	100.0	27.1	5.9	17.6	5.9
Climate sc.	86	22	249	29	14	21	23	386	9	26	11
percentage	22.3	5.7	64.5	7.5	3.6	5.4	6.0	100.0	2.3	6.7	2.8
Health	4	1	11	9	2	7	5	9	22	5	3
percentage	18.2	4.5	50.0	40.9	9.1	31.8	22.7	40.9	100.0	22.7	13.6
Engin.	22	3	64	9	5	7	15	26	5	88	5
percentage	25.0	3.4	72.7	10.2	5.7	8.0	17.0	29.5	5.7	100.0	5.7
Econ. sc.	3	4	14	5	6	10	5	11	3	5	21
percentage	14.3	19.0	66.7	23.8	28.6	47.6	23.8	52.4	14.3	23.8	100.0

Table shows frequencies and percentages for overlaps of disciplines, multiple answers were allowed (n = 1232).

### Perceived properties of open data

**Fig 2:**
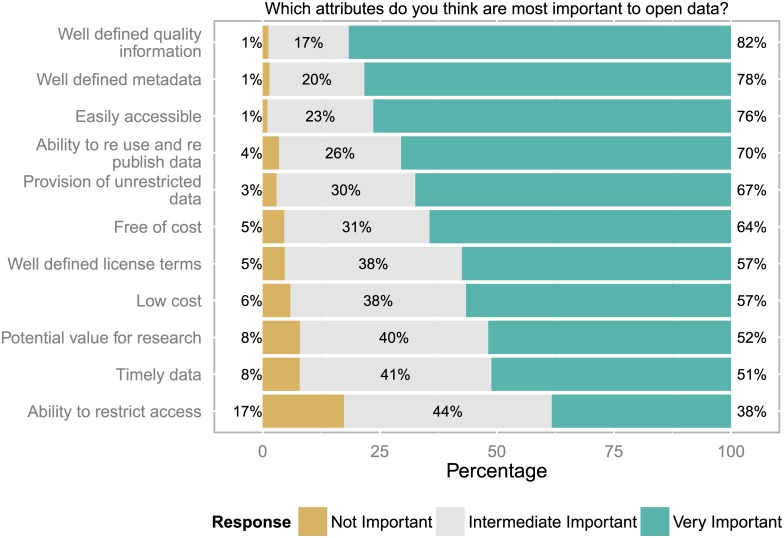
Perceived properties of open data. The ability to assess the quality, to select based on metadata, and to easily access and (re)use the data were rated as most important (n = 944 to 973 responses).

Instead of providing a definition of “open data” the survey assessed user perceptions associated with the term, based on their understanding of it (compare Fig 2). The answers to the question “Which attributes do you think are most important to open data?” highlight the importance of information which enables users to assess the quality of the data, to select based on metadata, and to easily access and reuse the data. The ability to restrict access was lowest in the ranking of desirable attributes, which fits with the intuitive idea of openness. However, nearly 2/5 of all respondents still considered the option to restrict access as a very important attribute. This may be due to the perception that keeping control of the release date of data into the open is essential, or that for some data types mandatory openness is not an option.

### Access and licensing conditions

Respondents were asked to assess a range of licensing approaches regarding their usefulness for open data. The licenses offered were: Public Domain (Creative Commons CC0, Public Domain Dedication License PDDL), Attribution (Creative Commons CC-BY, Open Data Commons ODC-BY), Attribution-Share Alike (CC-BY-SA, Open Database License ODbL), Non-Commercial (CC-BY-NC), No Derivatives (CC-BY-ND), Open Government License, and if “Other” was chosen to specify the answer.

**Fig 3:**
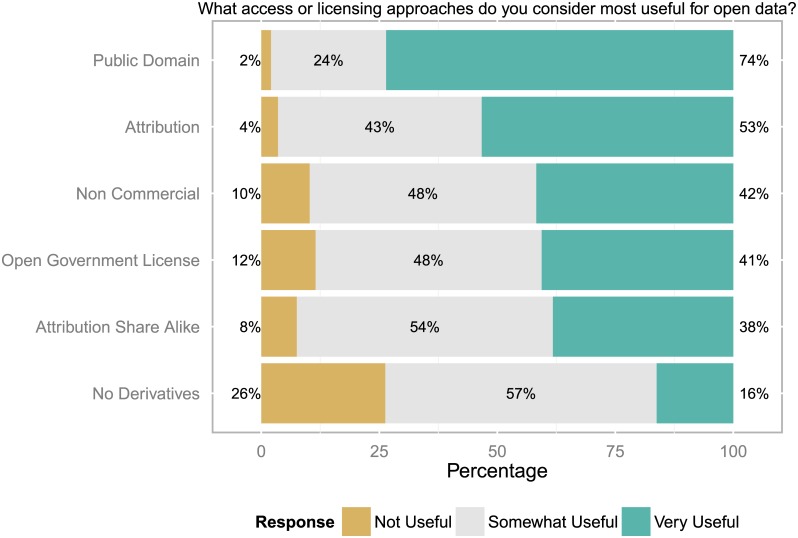
Views on licenses for open data. A “Public Domain” or “Attribution” license were considered most useful for open data (n = 712 to 820 responses).

“Public Domain” and “Attribution” licenses are considered very useful by the majority of respondents, which is in line with most recommendations by advocacy organizations, policy makers and research funders (compare Fig 3). Not surprisingly, “No Derivatives” is seen as least useful of all license options. Although references to definitions of these terms were provided in the survey some respondents still expressed difficulties in understanding license terms. However, although there seemed to be strong support for the most liberal licenses, some of this may have been due to the order in how the options have been offered in the survey (Public Domain, Attribution, Attribution Share Alike, Non Commercial, No Derivatives, Open Government License). The number of responses for these six options declined from 820 for the first to 712 for the last option.

Overall, 949 respondents shared their view on licenses (i.e. 75.7% of n = 1253). Among these, about one in four respondents (26%, 216 responses) expressed concerns about licenses which does not seem particularly high but might be due to inexperience or lack of interest in the topic. Explicit comments were provided by 166 respondents, ranging from considering licenses a burden, difficulties in understanding licenses, lack of standardisation, concerns about too restrictive licenses (in particular Non Commercial, Share Alike), concerns about misuse of data, attribution stacking (in relation to CC-BY) and issues of confidentiality (i.e. providing levels of open access).

Views on intellectual property issues were also assessed in terms of perceived barriers for publishing data as open data (cf. section “Barriers for publishing data as open data”).

### Guidelines for open data

Of those who provided an answer, only 23% (216 respondents) were aware of any guidelines for publishing data; all other 733 respondents did not seem to be aware of any specific guidelines. Overall, 175 respondents provided a wide range of examples of known guidelines, including (number of references indicated if >1, cf. S2 Appendix):

- licenses: Creative Commons (10 references), Open Data Commons (2 ref.)
- data policies (by funders, scholarly societies, etc.): European Commission (4 ref.), OECD (5 ref.), Research Councils UK (RCUK), Sunlight Foundation (5 ref.), American Geosciences Union (AGU), etc.
- repository/archive and e-infrastructures: PANGAEA (10 ref.), Dryad (6 ref.), DataONE (3 ref.), GBIF (4 ref.), CGIAR, European Vegetation Archive, etc.
- declarations, handbook, general information: Open Data Handbook (3 ref.), 5 star open data (3 ref.), Bouchout Declaration (2 ref.), etc.
- government data: US, France, Australia, Austria, etc.
- data publishing and data journals: Force11 Joint declaration on Data Citation Principles (2 ref.), Earth System Science Data, Pensoft, Wiley (2 ref.), Springer, Nature Publishing Group (2 ref.), European Geosciences Union (EGU) journals, etc.
- technical guides: W3C (9 ref.), DOI standard (ISO), etc.
- literature: e.g. Goodman et al (2014): Ten simple rules for the care and feeding of scientific data, PLoS Computational Biology, DOI: 10.1371/journal.pcbi.1003542

### Expectations about functionalities of infrastructures for open data

**Fig 4:**
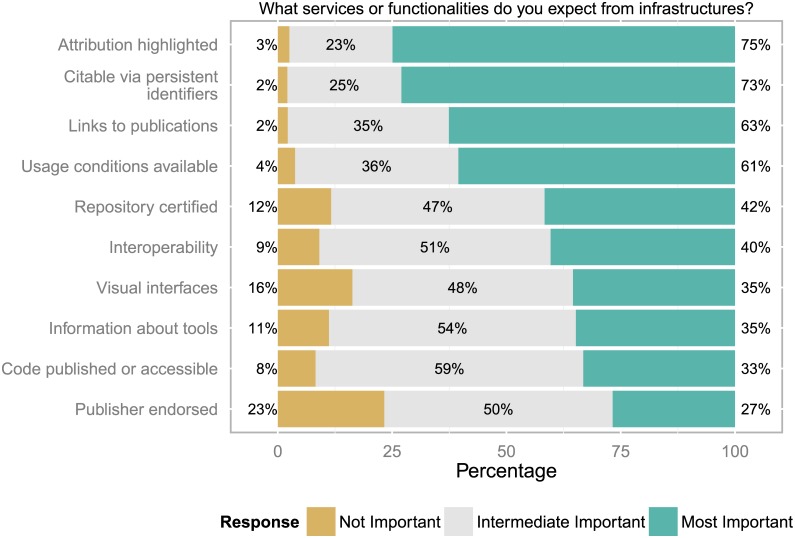
Expectations about functionalities of infrastructures. Core expectations of users of data infrastructure were that attribution information is provided and that data is citable (n = 890 to 911 responses).

The top four functionalities highlight the data user perspective (see Fig 4), i.e. that authorship and attribution information are highlighted, data are citable via persistent identifiers, links to publications are provided and restrictions, conditions and/or licensing information is communicated. Certification and interoperability were significantly less emphasized but still seen as most important by at least 40% of all respondents.

### Importance of open data for disciplinary communities

**Fig 5:**
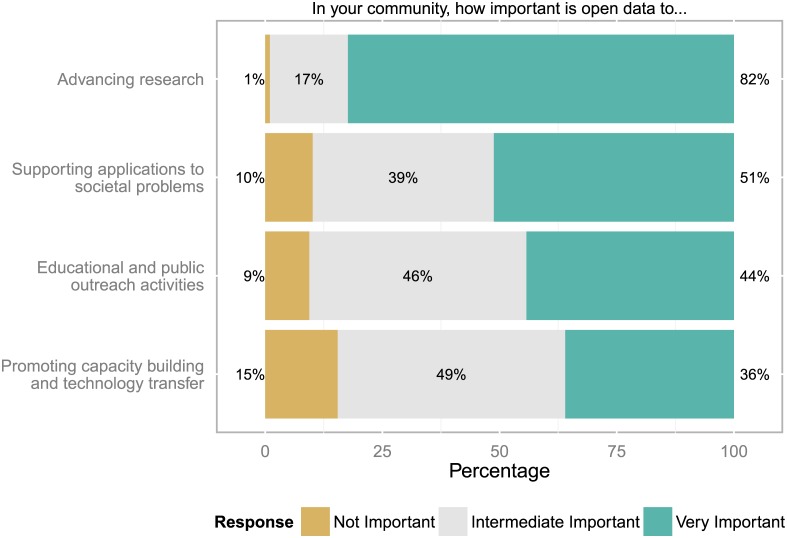
Importance of open data for disciplinary communities. Four out of five respondents highlighted that open data is crucial for advancing research (n = 853 to 878 responses).

When asked how important open data is for the disciplinary community, more than 4 out of 5 respondents highlighted that open data is very important for advancing research (compare Fig 5). Here it should be emphasised that participants to the survey might not be representative of the community as a whole and might also be more positive towards the topic than the average researcher. Of particular note, half of the respondents considered open data as important for supporting applications to societal problems.

### Motivators to publish data as open data

**Fig 6:**
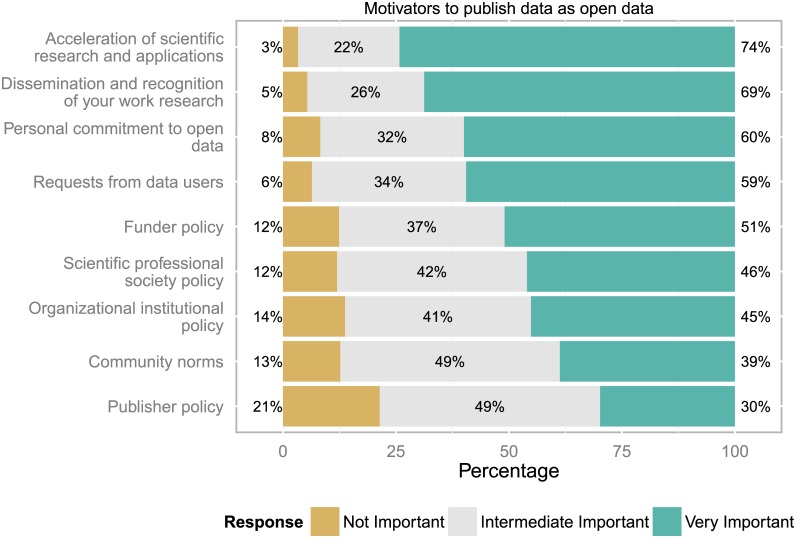
Motivators to publish data as open data. The commitment to publish data as open data seems to be driven by research-intrinsic motives, combining general and personal motivations (n = 834 to 861 responses).

The main desires to publish data as open data arose from research-intrinsic motives ranging from general considerations, i.e. the acceleration of scientific research and applications, to personal motivations, i.e. dissemination and recognition of research results, personal commitment to open data and requests from data users (see Fig 6).

Policies play a less strong but significant role, led by funder policies, and followed by scientific/professional society policies and organizational/institutional policies. Publisher policies were seen as less important but still recognized with some importance by almost 80% of all respondents.

Some respondents provided additional information about other motivators (13 responses), e.g. rankings of data, citations, metrics and incentives for career advancement were preferred over policy and requirements, improved data usability, automation of data movement, processing and annotation.

The order of the top four motivators was the same for all three subgroups of data professionals (data user, data provider, data manager) and there are only small differences in their attitudes to the motivators. However, data managers’ personal commitment to open data seems to be higher (70% very important, 24.3% intermediate important, 5.7% not important) than those of data users (59.9%, 33.7%, 6.4%) and data providers (61.1%, 31.5%, 7.3%).

From a two-sample Mann-Whitney-Wilcoxon (MWW) test applied to data managers vs. all other data professionals (i.e. all data users and data providers which are not simultaneously data managers), we can reject the null hypothesis that data managers and all other data professionals have the same attitude towards motivators at the 0.1% percent level (p = 0.0003, cf. Table 6). A one-way MANOVA test led to the same result (with slightly different p-values).

**Table 6:** Motivators for data managers vs. all other data professionals.

Motivator	n	low	interm.	high	p-value
Acceleration of scientific research and appl.	235	2.15	22.32	75.54	0.5318
Dissemination and recogn. of your work	231	3.48	24.35	72.17	0.1487
Personal commitment to open data	232	5.65	24.35	70.00	0.0003***
Requests from data users	231	7.66	33.19	59.15	0.7284
Funder policy	230	15.22	32.17	52.61	0.9979
Organizational institutional policy	231	15.09	37.93	46.98	0.8039
Scientific professional society policy	230	13.85	39.83	46.32	0.8076
Community norms	233	12.55	45.45	41.99	0.3322
Publisher policy	230	20.78	47.19	32.03	0.4843

At a personal level, data managers who contributed to the survey were significantly more committed to open data than all other data professionals (p-values based on a two-sample Mann-Whitney-Wilcoxon test, Levels of significance:

*: p<0.05,

**: p<0.01,

***: p<0.001.).

When comparing motivators across employment areas based on a one-way MANOVA, followed by one-way ANOVA tests and a post-hoc Tukey analysis only one difference was significant: respondents from academia expressed a stronger motivation by publisher policies than respondents working at government organizations (p = 0.002).

### Barriers for publishing data as open data

**Fig 7:**
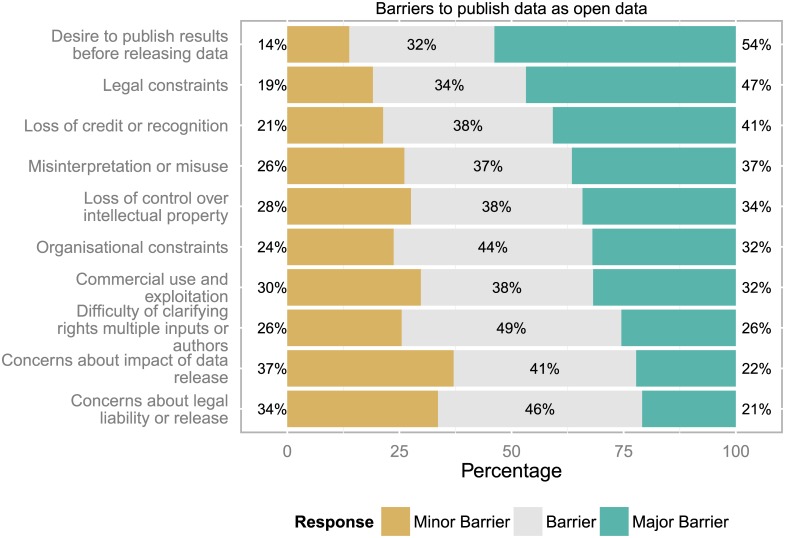
Barriers across countries. The release of data was seen as a secondary step compared to publishing results (n = 825 to 854 responses).

Overall, the most important barriers for publishing data as open data were the desire to publish results before releasing data, legal constraints, loss of credit or recognition and possible misinterpretation or misuse (see Fig 7). Concerns about legal liability for data or release of data were least-pronounced.

Some respondents provided information about other perceived barriers (28 responses), e.g. the size of data, laziness, lack of funder requirements to publish data but also if too many data policy apply, lack of attribution and credit, difficulty of using standards and the amount of time or costs that it takes to properly document the data so that it is useful for others.

The desire to publish results before releasing data was somewhat more prevalent at early stages of a research career. Using three age clusters 62% of the youngest cohort of respondents perceived the desire to publish results before releasing data as a major barrier vs. 51 and 49% of respondents of the other two age groups (35 and younger, 36–50, 51 and older, cf. Tables 7 and 8).

**Table 7:** Age groups (clustered).

Age group	Frequency	Percentage
20–35	397	31.8
36–50	528	42.3
51+	322	25.8

Table shows frequencies and valid percentages for each age group, derived from the original age groups (n = 1247).

**Table 8:** Publishing before releasing data—by age groups.

Age group	Minor Barrier	Barrier	Major Barrier	n
20–35	12.2 (30)	26.02 (64)	61.79 (152)	397
36–50	14.08 (49)	34.48 (120)	51.44 (179)	528
50+	15.04 (34)	36.28 (82)	48.67 (110)	322

Table shows percentages and frequencies for each age group.

**Fig 8:**
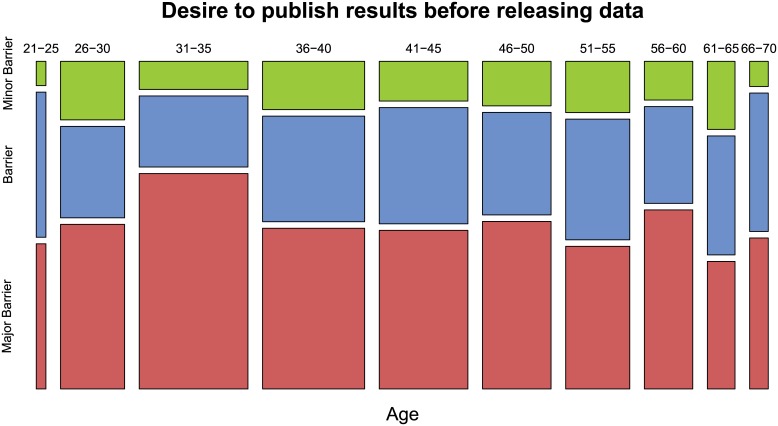
Desire to publish before releasing data by age groups. The willingness to share data share varies across age groups, the 31–35 year-olds expressed a significantly higher desire to publish results before releasing data. Due to the very small number of respondents in some categories the plot does not display all age groups (age < = 20 and >70 are not shown).

A Pearson’s Chi-squared test verifies that there is a significant difference in the attitudes of the three age groups although not very strong (p = 0.045). A one-way ANOVA test followed by a post-hoc Tukey analysis on the three age clusters reveals that the up to 35-year-olds and the 51+ year-olds differ significantly (p = 0.042). On the finer level, when we compare the group of 31–35 year-olds with all other age groups there is a stronger difference: Based on a Wilcoxon rank sum test the null hypothesis can be rejected at the 0.01% level (M = 40259.5, p<0.001, also compare Fig 8). Moreover, respondents of age 20–35 also expressed significantly higher concerns about the impact of data release (p = 0.024) compared to respondents of age 51 and older.

When comparing data managers’ attitudes towards barriers for publishing data as open data with those of respondents in other data professional roles based on a one-way MANOVA test no significant differences could be found (p = 0.089).

No significant differences towards barriers could be found when comparing the employment areas of respondents.

### Discovery of open data

**Fig 9:**
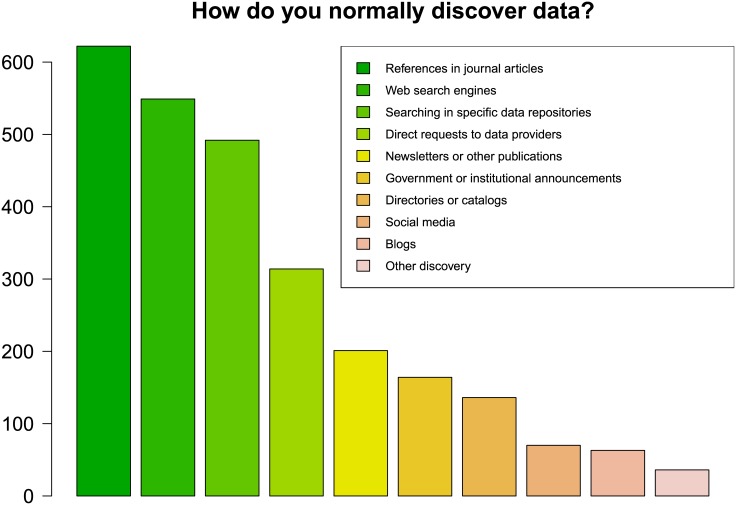
Discovery of data. References in journal articles, web search engines and data repositories were identified as the most common discovery routes (n = 774 respondents selected at least one option).

Of all respondents, 779 individuals provided information about their discovery routes to open data (see Fig 9 and Table 9).

**Table 9:** Discovery routes.

Discovery route	Frequency	Percentage
References in journal articles	622	79.8
Web search engines	549	70.5
Searching in specific data repositories	492	63.2
Direct requests to data providers	314	40.3
Newsletters or other publications	201	25.8
Government or institutional announcements	164	21.1
Directories or catalogues	136	17.5
Social media	70	9.0
Blogs	63	8.1
Other discovery	36	4.6

Table shows frequencies and valid percentages for each discovery route, multiple answers were allowed (n = 779).

Note that respondents could select multiple options. For these, discovery of open data primarily takes place via references in journal articles (79.8%), web search engines (70.5%) and searching in specific data repositories (63.2%). Moreover, 40.3% of all respondents use direct requests to data providers. Newsletters or other publications, government or institutional announcements and directories and catalogues play a less important role but are still recognized by about 1/4 to 1/5 of all respondents (25.8% to 17.5%). Social media and blogs seem only be used by less than 1/10 of all respondents (8.1% for blogs). In addition, 4.6% of all respondents provided information about other discovery routes in descending order of frequency (some overlap with the options above), e.g. personal contacts with colleagues and scientific communities, presentations at conferences and workshops, larger projects / programs, (open) data journals, mailing lists, data centres and data portals and books.

### Data archives

Data archives can be used for different purposes, for discovery of data and/or for publishing data, and the survey did not distinguish between these. Respondents were also asked to recommend leading examples inside or outside of their community.

For finding or accessing data a wide variety of data repositories and data portals, but also general search engines, were mentioned, e.g. (roughly ordered based on a word cloud): Global Biodiversity Information Facility (GBIF), Pangaea, Dryad, Genbank, Google, National Center for Biotechnology Information (NCBI), National Oceanic and Atmospheric Administration (NOOA), NASA, European Centre for Medium-Range Weather Forecasts (ECMWF), U.S. Geological Survey (USGS).

For publishing data, the following data repositories were most popular (roughly ordered based on a word cloud): Pangaea, Dryad, NCBI, e.g. Genbank, European Nucleotide Archive (ENA), NOOA, e.g. paleoclimatology data, GBIF, Figshare, GitHub, Carbon Dioxide Information Analysis Center (CDIAC), British Atmospheric Data Centre (BADC). Although data can not directly be published through GBIF, as it is an international data infrastructure based on a network of nodes, it was mentioned several times.

More generally, several respondents pointed out that they publish data via institutional or organisational data repositories and/or via journals.

Leading examples as suggested by the respondents included the following data repositories (roughly ordered based on a word cloud): NASA, Dryad, NOAA, GBIF, Pangaea, Figshare, data.gov.au. Again, as noted above GBIF does not quite fall into the data repositories category but was pointed out as a leading example by several respondents.

The outcomes of these questions have been shared with re3data.org and Databib, the registries of research data repositories which have recently merged their collections.

### Burden when accessing and reusing data

**Fig 10:**
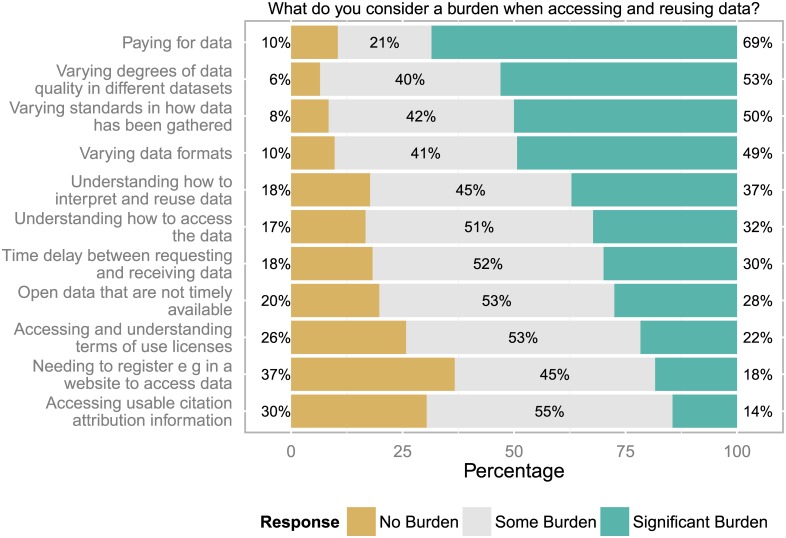
Burden when accessing data. Paying for data as well as varying data quality, standards and formats were considered least acceptable when accessing data (n = 687 to 731 responses).

Paying for access was considered the most significant burden when accessing and reusing data (see Fig 10). It cannot be assessed how frequently paying actually takes place as this question was not included in the survey. Further, about half of all respondents considered the varying degrees of data quality in different datasets, varying standards in how the data has been gathered and varying data formats as an obstacle. At least one third of respondents considered it an issue how to understand, how to access, interpret and use the data. At the lower end the issue of how to access usable citation and attribution information was still seen as some burden for 70% of all respondents. But the need to register before accessing the data did not seem to present a significant burden.

### Wishes regarding open data

Several desires regarding access to data were expressed. Wishes range from improved access to climate data from specific countries, e.g. China, India, Russia, Asia, South America, France, and developing countries; the support of long-tail research datasets via repositories (incl. institutional); access to private-sector and economical data (e.g. food trade and production data, insurance sector data, energy consumption data). Moreover, the lack of community standards was mentioned, in e.g. oceanography. One individual highlighted that “more could be done for the expansion of access to freely available older references, esp. for in-copyright publications, e.g. via OCR”. In addition, concerns were expressed regarding the needed resources: “addressing all the attributes that data users would like to see is impossible with current funding and resources. Efforts must not be at the expense of existing programs that are already underfunded. New and substantial resources are needed to make this happen.”

### Disciplinary differences

When considering disciplinary differences, it should be taken into account that for some research areas the number of non-trivial responses was rather low (e.g. social sciences about 40, agricultural and veterinary sciences about 35, chemical sciences about 30, health sciences and economical sciences about 10 to 15 non-trivial responses each).

#### Motivators by discipline

Motivations for sharing data as open data among earth, climate and atmospheric sciences were well-aligned with the average for all disciplines across the survey. These research areas share the top two motivators with the average attitude but ranked requests from data users third. Engineers and computer scientists expressed a slightly higher personal commitment to open data (2/3 considered it as very important and more than another 1/4 as intermediate important) while this was somewhat less so for economics (with rank 8 in the order of motivators, however, nearly 4/5 of all respondents considered this motivator as intermediate or very important).

Policies were considered as more important by the social sciences, with funder policy ranking second and institutional policy ranking third. Among the chemical sciences funder policy ranked first. In economics, scientific professional society policy ranked first and funder policy ranked second, both considered very important by 80% of all respondents. All respondents from economics considered community norms as important (with one half of respondents accounting for intermediate and another half for very important).

Publisher policies were seen as the least important motivator by the majority of disciplines but were considered to have some degree of importance by at least 4 out of 5 respondents, ranging from 75% (physical sciences) to 93% (economics).

The survey design did allow respondents to select more than one disciplinary area. Due to resulting overlaps we restrict ourselves to a two-sample comparison of all respondents from one selected discipline with all respondents from the complementary sample, i.e. all respondents which did not selected the disciplinary area. A one-way MANOVA test with follow-up univariate one-way ANOVAs and a Tukey’s post-hoc analysis confirms some of the observations described above. We report in this article only differences that were statistically significant.

Respondents from the biological sciences considered requests from data users a less strong motivator than the comparison group (p = 0.021, based on 179 responses from biological sciences vs. 677 responses from other disciplines). However, publisher policy was seen as a stronger motivator for the biological sciences (p<0.001, 179 responses from biological sciences vs. 666 responses from other disciplines).

No specific motivators were statistically significant for respondents from the physical sciences, chemical sciences, earth sciences and environmental sciences, agricultural and veterinary sciences, social sciences, computer sciences, climate and atmospheric sciences, health sciences, economical sciences, and other sciences.

#### Barriers by discipline

The desire to keep control of their data seemed to be more prevalent for some disciplines. Across all disciplines, about every second respondent considered the desire to publish as a major barrier to releasing the data as open data (slightly less so in engineering and economics, and significantly higher in the biological and chemical sciences). This was most pronounced in the chemical sciences (over 4 out of 5 of this subgroup of respondents (85%)) and least pronounced in engineering (about 2 out of 5 of this subgroup of respondents (45%).

In economics and engineering the desire to publish first was not the most prominent barrier, but legal constraints with 67% and 50%, respectively. The social sciences also seemed to be rather concerned about legal and organisational constraints, with 48% and 42% seeing this as a strong burden and even 91% and 86% as at least a burden.

Legal constraints were felt particularly strongly in economics with 67% seeing this as a strong barrier. In the health sciences only 38% saw legal constraints as a strong barrier (lowest across all disciplines) but an additional 54% as a barrier.

In the health sciences commercial use and exploitation ranked second as a major barrier (46% major barrier, 38% barrier) and organisational constraints ranked third (46% major barrier, 46% barrier).

A one-way MANOVA test with follow-up univariate one-way ANOVAs and a Tukey’s post-hoc analysis confirms some of the observations as described above. We report differences that were statistically significant. Overall, respondents from the physical sciences were less concerned about legal liability for data or the release of data (p = 0.015, 105 responses from physical sciences vs. 734 responses from other disciplines). Respondents from the chemical sciences were more concerned than the complimentary group about misinterpretation and misuse (p = 0.001, 30 responses from chemical sciences vs. 825 responses from other disciplines) and had a stronger desire to publish results before releasing data (p = 0.005, 27 responses from chemical sciences vs. 798 responses from other disciplines). Respondents from biological sciences seemed to feature stronger concerns about organisational constraints (p = 0.006, 173 resp. from biological sciences vs. 652 other responses) and the impact of data release (p<0.001, 179 vs. 654 resp.), and felt a stronger desire to publish before releasing data (p = 0.007, 173 vs. 652 resp.). Respondents from computer sciences were more concerned about the impact of data release (p<0.001, 57 from computer sciences vs. 776 other resp.), and slightly more concerned about legal constraints (p = 0.041, 58 vs. 795 resp.) and legal liability for data or release of data (p = 0.038, 57 vs. 782) (for the last two barriers a post-hoc Tukey test did not indicate a significant difference between the two samples). Respondents from climate and atmospheric sciences (846 responses) were significantly less concerned about commercial use and exploitation (p = 0.016, 279 responses from climate/atmosph. sciences vs. 570 responses from other disciplines), the misinterpretation or misuse of data (p = 0.003, 280 vs. 574 resp.), legal liability of data or release of data (p = 0.014, 276 vs. 563 resp.), and the impact of data release (p = 0.007, 271 vs. 621 resp.). Similarly, respondents from engineering (88 responses) were more concerned than the complementary group about organisational constraints (p = 0.012, 61 resp. from engineering vs. 790 resp. from other disciplines), commercial use and exploitation (p = 0.033, 51 vs. 798 resp.), legal liability for data or release of data (p = 0.007, 61 vs. 778 resp.) and the impact of data release (p = 0.002, 62 vs. 771 resp.).

For the earth and environmental sciences, social sciences, agricultural and veterinary sciences, health sciences, economics and other disciplines no significant differences compared to the respective complementary sample of respondents could be found.

### Selected observations by country and geographical region

**Fig 11:**
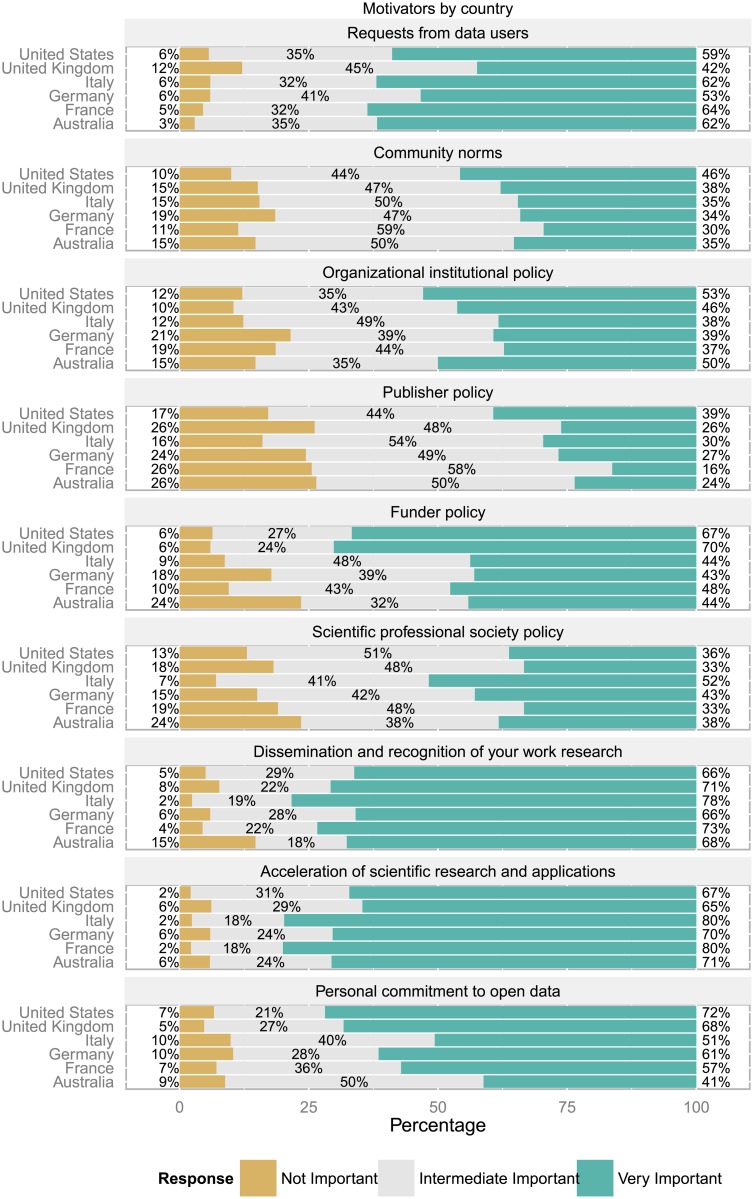
Motivators by country. Across all countries the acceleration of scientific research and applications, and dissemination and recognition of a researcher’s work were important reasons for sharing data. Funder policies as a motivator stood out in the UK and the U.S.

Let us now consider all countries for which at least 45 responses are available in the survey, resulting in six countries (United States, Germany, Italy, United Kingdom, France and Australia) where at least 30 non-trivial answers were provided to all topics of the question about what motivates to publish data as open data (compare Fig 11).

Comparing these six countries, requests from data users seemed to be of high relevance for 3 out of 5 researchers for most countries (France, Italy Australia, U.S., ranging from 64 to 59%), while only for every second (53%) from Germany and 2 out of 5 (42%) for the UK. The assessment of the importance of community norms seemed to be less pronounced with the U.S. (46%) at the higher end and France (30%) at the lower. Organizational institutional policy was seen as very important by every second respondent in the U.S. and Australia, while less so in all other countries (from 46% in the UK to 37% in France). Publisher policies were most pronounced as motivators in the U.S. (39%) and least so in France (16%). Funder policies seem to be much more established as a motivator for publishing data in the UK and the U.S. (70% and 68% respectively), while considered very important by almost every second respondent in France, and by 2 out of 5 in Australia, Italy and Germany. Scientific professional societies provide guidance to researchers too and their importance as motivators was recognized by every second respondent from Italy, otherwise closer to 2 out of 5 (Germany, Australia) or every third respondent in all other countries. Dissemination and recognition of one’s own research work was seen as very important by at least 2/3 of all respondents across all considered countries, from 66% in the U.S. and Germany to 78% in Italy. Acceleration of scientific research and applications was considered a strong motivator for 4 out of 5 respondents in Italy and France, and for about 2/3 for respondents from all other countries. Personal commitment to open data as a motivator seems to be somewhat stronger in the U.S. and the UK (72% and 68% resp.) compared to 41% in Australia to 61% in Germany.

In order to assess which of these differences are statistically significant, a one-way multivariate analysis of variance (MANOVA), followed by corresponding ANOVA tests and a Tukey post-hoc analysis was conducted on the set of motivators (cf. Table 10).

**Table 10:** Motivators by Country.

Item	Australia		France		Germany		Italy		UK		U.S.		ANOVA
	M	SD	M	SD	M	SD	M	SD	M	SD	M	SD	F;p
M1	2.59	0.56	2.59	0.58	2.47	0.61	2.56	0.61	2.30	0.68	2.53	0.60	1.63; 0.151
M2	2.21	0.69	2.18	0.62	2.16	0.71	2.19	0.69	2.23	0.70	2.36	0.66	0.84; 0.519
M3	2.35	0.73	2.19	0.73	2.18	0.76	2.26	0.67	2.36	0.67	2.41	0.70	0.97; 0.439
M4	1.97	0.72	1.91	0.65	2.02	0.72	2.45	0.63	2.00	0.73	2.22	0.72	1.53; 0.178
M5	2.21	0.81	2.38	0.66	2.25	0.74	2.14	0.67	2.64^a^	0.60	2.60^b^	0.61	3.99; 0.001**
M6	2.15	0.78	2.14	0.72	2.28	0.71	2.35	0.64	2.15	0.71	2.23	0.66	0.98; 0.429
M7	2.53	0.75	2.69	0.56	2.60	0.60	2.76	0.48	2.63	0.63	2.61	0.58	1.06; 0.38
M8	2.65	0.60	2.78	0.47	2.64	0.59	2.77	0.47	2.58	0.61	2.65	0.52	1.38; 0.232
M9	2.32	0.64	2.50	0.63	2.51	0.68	2.41	0.67	2.63	0.58	2.65^c^	0.60	3.72; 0.003**

Table shows mean agreement and standard deviation for all countries with at least 45 responses. The p-values are based on univariate ANOVA tests within omnibus MANOVA (MANOVA: F(9, 466) = 1.81; p = 0.001***). Levels of significance:

*: p<0.05,

**: p<0.01,

***: p<0.001.

Tukey’s post-hoc analysis:

^a^: The UK differs significantly from the Germany and the Italy,

^b^: The U.S. differ significantly from the Germany and the Italy,

^c^: The U.S. differ significantly from Italy and Australia.

Motivators: M1: Requests from data users, M2: Community norms, M3: Organizational institutional policy, M4: Publisher policy, M5: Funder policy, M6: Scientific professional society policy, M7: Dissemination and recognition of your work research, M8: Acceleration of scientific research and applications, M9: Personal commitment to open data.

Regarding the perception of motivators, the difference of mean agreement across the six countries varies significantly (F(9, 466) = 1.81; p<0.001). ANOVA tests combined with Tukey’s post-hoc analysis show that funder policy plays a significantly stronger role in the UK and the U.S. compared to Germany and Italy. In addition, personal commitment to open data was a significantly stronger motivator for respondents from the U.S. compared to those from Italy and Australia.

Regarding the perception of barriers, a one-way MANOVA shows that mean agreement across the six countries under consideration varies significantly (F(10, 453) = 1.99; p<0.001, cf. Table 11).

**Table 11:** Barriers by Country.

Item	Australia		France		Germany		Italy		UK		U.S.		ANOVA	
	M	SD	M	SD	M	SD	M	SD	M	SD	M	SD	F;p
B1	2.50	0.62	2.09	0.80	2.39	0.71	2.35	0.74	2.29	0.78	2.08^a^	0.77	4.78; 0.0003***
B2	2.18	0.80	2.20	0.78	2.00	0.76	2.13	0.78	1.98	0.73	2.06	0.70	0.56; 0.727
B3	2.09	0.90	2.02	0.81	2.01	0.79	2.24^b^	0.73	2.00	0.80	1.81	0.75	3.06; 0.01**
B4	1.85	0.67	1.93	0.78	2.13	0.84	2.34^c^	0.72	1.97	0.76	1.86	0.81	4.4; 0.001***
B5	1.91	0.87	2.00	0.76	1.96	0.80	2.23	0.73	2.09	0.77	2.08	0.76	2.13; 0.06
B6	2.03	0.76	2.19	0.74	2.16	0.74	2.25	0.75	2.15	0.78	2.11	0.77	0.4; 0.851
B7	1.82	0.72	1.88	0.66	2.09	0.73	2.17	0.74	1.91	0.72	1.82^d^	0.65	2.96; 0.012*
B8	1.82	0.76	1.60	0.63	1.85	0.70	2.00	0.69	1.83	0.72	1.70	0.72	2.2; 0.053
B9	1.68	0.73	1.77	0.74	1.83	0.72	2.09	0.73	1.83	0.79	1.78	0.77	1.97; 0.081
B10	2.06^e^	0.78	2.23	0.81	2.52	0.68	2.29	0.75	2.45	0.64	2.38	0.72	3.28; 0.006**

Table shows mean agreement and standard deviation for all countries with at least 45 responses. The p-values are based on univariate ANOVA tests within omnibus MANOVA (MANOVA: F(10, 453) = 1.99; p = 5.011e-05***). Levels of significance:

*: p<0.05,

**: p<0.01,

***: p<0.001.

Tukey’s post-hoc analyis:

^a^: The U.S. differ significantly from Australia and Germany;

^b^: Italy differs significantly from the U.S.;

^c^: Italy differs significantly from the U.S. and Australia;

^d^: The U.S. differ significantly from Germany and Italy;

^e^: Australia differs significantly from Germany.

Barriers: B1: Legal constraints, B2: Organisational constraints, B3: Commercial use and exploitation, B4: Loss of control over intellectual property, B5: Misinterpretation or misuse, B6: Loss of credit or recognition, B7: Difficulty of clarifying rights multiple inputs or authors, B8: Concerns about legal liability for data or release of data, B9: Concerns about impact of data release, B10: Desire to publish results before releasing data.

The corresponding univariate ANOVA tests and a Tukey’s post-hoc analysis reveal several significant differences: legal constraints were seen as a significantly higher barrier by respondents from Australia and Germany compared to respondents from the U.S. Moreover, there was a significantly stronger urge by Italian respondents towards commercial use and exploitation compared to respondents from the U.S. Loss of control over intellectual property was perceived as a significantly higher concern for respondents from Italy than for respondents from the U.S. and Australia. Difficulties of clarifying rights vis-à-vis multiple inputs or authors was seen as a significantly stronger barrier by respondents from Italy and Germany in comparison with respondents from the U.S. The desire to publish results before releasing data was significantly stronger for respondents from Germany compared to those from Australia.

In addition, attitudes can be compared by geographic regions. For this MANOVA/ANOVA analysis countries were clustered according to continents (cf. Table 12). As there were very few responses from Africa (n = 14) these responses have not been considered in the comparison across regions.

**Table 12:** Countries by continent.

Continent	Frequency	Percentage
Europe	797	63.9
North America	216	17.3
Asia	130	10.4
Australia / New Zealand	53	4.2
South America	38	3.0
Africa	14	1.1

Table shows frequencies and valid percentages for each regional category (n = 1248).

Respondents from Asia (p = 0.002) and Europe (p = 0.023) expressed significantly stronger concerns about legal constraints than respondents from North America. Regarding commercial use and exploitation respondents from Latin America (p<0.001), Asia (p<0.001) and Europe (p = 0.012) expressed significantly stronger concerns than respondents from North America. In addition, respondents from Latin America are also more concerned than respondents from Europe (p = 0.02). Respondents from Asia were significantly more concerned about the loss of control over intellectual property than respondents from both North America (p<0.001) and Australia / New Zealand (p = 0.039). Moreover, European respondents expressed a significantly stronger agreement with this statement than respondents from North America (p = 0.029). Respondents from Asia were more concerned about misinterpretation or misuse than respondents from Europe (p = 0.039). Regarding difficulties of clarifying rights vis-à-vis multiple inputs or authors respondents from Asia expressed significantly higher concerns than those from North America (p = 0.001). Respondents from Asia were significantly more concerned about legal liability for data or the release of data than respondents from Europe (p<0.001), North America (p<0.001), Australia and New Zealand (p = 0.001). Similarly, Asian respondents expressed significantly higher concerns about the impact of data release compared to respondents from Europe (p<0.001), North America (p<0.001), Australia and New Zealand (p = 0.017). The desire to publish before releasing data was significantly higher by respondents from Asia compared to both those from Australia and New Zealand (p = 0.008) and Europe (p = 0.028).

With regard to motivators for publishing data as open data, publisher policies were seen as a significantly stronger motivator by respondents from Asia compared to those from Europe (p = 0.006). Respondents from Asia also considered scientific professional society policy as a significantly stronger motivator than respondents from Europe (p = 0.03), North America (p<0.001), Australia and New Zealand (p = 0.009).

## Discussion

Scientific research nowadays is changing to a paradigm of more data intensive science and collaboration compared to research in the past [3]. The growth of extensive computing facilities and access to large datasets open major opportunities for researchers to address complex, multi-dimensional questions dealing with urgent environmental issues, such as climate change, natural resource alterations, depletion, and biodiversity loss, which allow for model-field-laboratory inter-comparisons and require multi-, inter- and trans-disciplinary analyses [4–7]. However, scientific communities are now confronted with a broad suite of technical issues associated with data management [7]; in particular access to a wide variety of information brings with it greater complexity, transparency, integrity and interpretation problems [6]. This demands a stronger community awareness about issues of data archiving and sharing, which is strongly reflected by the supportive responses to our survey.

In our discussion of the results of this survey, we have assumed that the distribution via mailing lists primarily reached scientists who are already somewhat familiar with the topic, e.g. the high response rate via the Copernicus Publications mailing list points in this direction. This, of course, also holds for other surveys on similar topics, e.g. Tenopir et al [8] state that “a bias based on interest, familiarity, personal relevance, or favorable feelings toward the topic should not be underestimated”. Tenopir et al. report an increased acceptance of, and willingness to participate in, data sharing, as well as a rise in actual data sharing activities, and note that these are changes that have occurred in the past 3–4 years.

In our survey, the majority of respondents (98.3%, n = 1232 of 1253 responses) revealed their field of scientific research, with the majority working in academia (70%, 878 responses); however, also a total of 25% (315 responses) claimed to be data managers, who most likely have different perspectives towards open data concerning access, licensing, barriers and motivation. The DataONE survey [9] and the Kratz and Strasser’s [10] survey responses came to 81% and 85% from academia and thus, most likely reflect a slightly stronger scientific attitude than our survey does. The survey by the Expert Advisory Group On Data Access [11] in the United Kingdom mainly targeted principle investigators. The Tenopir et al. surveys collected information about primary subject disciplines but did not differentiate between data managers and researchers [3, 8].

Our largest response sets were based in the earth and environmental sciences, climate and atmospheric sciences, and biology. The DataONE surveys [3, 8, 9] concentrated on researchers from the environmental sciences and ecology. Kratz and Strasser’s survey [10] on data publication had a bias towards biological sciences. The EAGDA survey [11] featured a bias towards social sciences and epidemiology. The majority of responses in our survey came from central Europe and the U.S., while the DataONE surveys and the Kratz and Strasser survey all had a stronger focus on scientists based in the U.S., and the EAGDA survey (2014) was focusing on UK scientists only. In addition, there are several other institute or community specific surveys; however, the broader context for comparison on an international scale was the main scope for the Belmont Forums’ survey, therefore only the surveys conducted with a broader scope will be taken into account for the discussion.

Currently, different scientific communities feature diverse degrees of maturity in data archiving practices and standards (e.g. [3, 8, 12, 13]). For example, standardized data archiving in geophysics, astronomy and genomics is well established via the World Data Systems (WDS) and the International Nucleotide Sequence Database Collaboration (INSDC), while data archiving for many other kinds of data is just emerging. However, in our analysis the overall acceptance and motivation to store and use open data was comparatively high throughout all discipline communities, ages, employment areas, and nationalities and render concerns about empty archives unnecessary [14]. Although our survey did not explicitly ask if respondents were willing to share data, 98% claimed publishing of open data to be important in their community to advance research. This high level of support is similar to findings by Enke et al [15] but stands in contrast to the findings of Tenopir et al [3] where 46% of the responding scientists reported they were not willing to make their data electronically available to others. However, the attitudes reported in that survey changed over time, as 3–4 years later, researchers indicated significantly more willingness to share and reuse data compared to the base-line survey [8]. Discrepancies in responses between our survey and Tenopir et al.’s surveys might be due to different groups of respondents, different scientific research communities, or a change in attitude over time [3, 6, 8, 16]. Many authors have promoted the benefits of data sharing [3, 10, 17–23] which might have impacted the research community which responded to our survey as well as others. This might trigger some attitudinal changes towards open data archiving and use in the future. For a comprehensive overview of current discussions and the issues involved in data publishing also compare [24].

Discussions about how to define open data have led to quite different results (e.g. [25–28]). An assessment of user perceptions associated with open data confirms varying attitudes while the common idea behind the term seems to be well understood. A majority of the respondents claimed that well-defined quality information and metadata are the most important attributes of open data for them, next to easy access to data and the ability to use and re-use data.

Licensing of open data was considered as useful and concerns about licensing data did not seem to be particularly strong) possibly due to limited interest in this question). However, when it comes to perceived barriers to the release of data as open data IPR-related barriers play a significant role (compare section “Access and licensing conditions”).

Regarding guidance on open data, in 2007 the OECD Recommendations defined principles and guidelines for access to research data which the member countries were expected to implement [29]. Despite this there are still very few guidelines at the national level (e.g. [30–32]); nonetheless, guidelines for open data are provided by several funders and scholarly societies, data repositories and archives, as well as data journals. However, less than $\frac{1}{4}$ of our respondents were aware of any guidelines or recommendations of publishing data as open data; pointers to several guidelines and recommendations were provided. Awareness of guidelines among respondents who identified themselves as data managers was slightly higher compared to other data professional roles (data managers: 35%, 86 of 248 respondents; data providers: 25%, 140 of 552 respondents; data users: 20%, 158 of 785 respondents). This seems to indicate that there is still only moderate awareness within the research community about recommendations regarding open data publication and re-use. Therefore, broader dissemination and communication efforts regarding the importance of, and available guidance on, data management is highly recommended.

Even with willingness to share data there are discrepancies with common practices, e.g. willingness to spend time and resources preparing and up-loading data (e.g. [33]). The main motivations for data sharing highlighted in our survey were accelerating research and applications, followed by scientific merits and recognition of data sharing efforts. In the PARSE.Insight survey of European scientists Kuipers and van der Hoeven [34] described the potential for re-analysis of existing data as the most important driver for the preservation of research data. Regarding potential author benefits, Piwowar and Vision [35] discovered a robust citation benefit from publishing data. UK life scientists in the RIN report [12] stated that “in a competitive environment, the willingness to share is subject to reservations, in particular to the control they have over the manner and timing of sharing”. Therefore, measures to increase support and trust seem to be most effective in motivating investigators to document and provide access to their data (cf. [7, 10, 36]). The EAGDA survey [11] states that currently there is very little, if any, formal recognition of data outputs in key assessment processes, funding decisions, or academic promotion (e.g. [7, 36]). This has been realized by some publishers, which are creating persistent links from articles to relevant datasets to support the scholarly record, which was highly acknowledged by researchers in the RIN report in 2008. However, recognition of data as core research outputs can nowadays also be achieved through initiatives like data journals and data citation indices (e.g., Web of Science Data Citation Index), as scientific merits and accelerating research and applications are still the main motivators to publish data as open data (cf. [37, 38]).

Of all respondents, 88% acknowledged that funder-endorsed data policies motivate them to publish data as open data. Therefore it seems that the acceptance of open data could be further enhanced through making open data archiving mandatory; this is currently not the case for funders in several countries. In a recent analysis Fecher et al. 2015 [39] addressed the issue that “research policies that better incentivise data sharing are needed to improve the quality of research results and foster scientific progress”. However, several research policy makers world-wide support and foster the accessibility of research data, e.g. the U.S. National Institutes of Health (NIH 2002, 2003), the National Science Foundation, and the European Commission [40].

According to our survey, the most common way to discover primary data was through references in journal articles. We assume that in the long run single data sets or articles will not be the main focus of interest; instead linked open data, content mining, both for metadata and data, mining and access to machines over a wide range of access will be of greater interest. This has already been recognized by publishers who consider and discuss primary data archives as potential future service area and sources of income. However, over 90% of the respondents considered paying for access and reuse of data a major or some burden and claimed that open data should be free or of low cost. This result confirms the findings of the RIN report [41] where the payment of fees was also named as a major obstacle.

In our survey the most important barrier for publishing data as open data was the desire to publish the results before releasing data. In the PARSE.Insight report Kuipers and van der Hoeven [34] described researchers’ concerns regarding legal issues and potential misuse of their data as major barriers for sharing research data, which was named as second most important barrier in our assessment. Further barriers to open data as identified by the analysis of Michener et al [9] included the lack of technical implementations for acknowledging data contribution and easy to use tools for data access, conversion and analysis. This is in concordance with the results of our survey which provides evidence that authorship and attribution information as well as citability via persistent identifiers and links to publications are considered as the most important functionalities of data infrastructures. Ethical concerns with regard to data sharing should also not be underestimated, as they already were brought up in the report from the Research Information Network [41] and they were the second most frequently mentioned constraint by 55% of respondents by the EAGDA survey in 2014 [10, 11, 42]. Establishing trust between parties in form of mutually acceptable intellectual property agreements might be difficult, but would certainly result in enhanced data sharing, productivity and the generation of useful, robust outcomes [6, 43–48]. In the survey by Michener et al [9] technical barriers were named as one major burden to data sharing due to the unavailability of data repositories and unity standards (e.g., metadata standards), with repositories and standards being also mentioned by a similar survey in Australia [6]. This however was not explicitly stated as a barrier in our analysis and might be due to the differences in survey respondents.

For data re-use, a majority of the respondents to our study claimed well-defined quality information and metadata as the most important attributes. These findings are in accordance with what Borgman [49] found: “that an effective data reuse is often too challenging for the individual scientists as individual datasets are often only accessible with different protocols and via different user interfaces”. Due to the underlying complexity this often demands expert knowledge in computer science [50], as research data cannot be regarded as general knowledge [39]. The RIN report as early as 2008 [12] recognized that “effective use of raw scientific data … may require access to sophisticated specialist tools and technologies, and high level programming skills”. Tenopir et al. [3] reported that nearly half of their respondents did not have any support or training, and they claimed “even if investigators desire to share their data, many feel that they lack the skills to do so effectively” (with similar conclusions cf. also [7, 51]). In Australia, Henty et al. [52] discovered that most researchers are willing to share their data, but they would like sharing to be easier. In particular, they seemed to be worried by the bureaucratic requirements related to data management. Earlier, a similar picture emerged in the UK [12] and in the PARSE.Insight survey [34], with a demand for simple tools to facilitate data sharing. However, Tenopir et al. [8] discovered that there has been a significant change in data management support, compared to the earlier investigation [3]. Several different initiatives have addressed the deficit of relevant skills, training and career structures for data management (e.g. [12, 53]). Bolukbasi et al. stated the training especially of early career scientists on methods and protocols for data management to be beneficial for the motivation of data sharing, as good habits will develop, which contribute to a culture of proactive data sharing and stewardship (e.g., [7, 36]) and Tenopir and co-workers even defined stronger incentives: “if support were to be offered as shared services, there is a strong provision that these should be customer-focused and should meet local circumstances and needs” [8].

Trusted data repositories could further ease this process by providing technical and administrative support for researchers, e.g. in form of easy-to-use tools that support integration, analysis, and visualization of data across diverse data types and standards, web interfaces for up- and download, ancillary benefits that can be derived from data mining and analysis tools, help desk support, and the ability to cite published data [9, 37, 54–56].

Already the EAGDA report [11] mentioned that this demands significant investments to support key data infrastructures, to serve the data community by meeting the long-term costs of data preservation, and developing user-friendly services that support researchers as far as possible. This need is especially pressing for highly acknowledged data repositories which do not have clear mandates for organizations and might be in the interest of commercial publishers in order to have a base for commercial services. To guarantee for the long-term that primary research data—mainly generated with governmental support—will still remain a public good requires sufficient and long-term financial commitment and support for sustainable infrastructures and high-quality (open) data stewardship [57], as data must be curated over the long term to maintain public access, provide backups, and updated to keep pace with changing technology [7].

Based on the findings of the survey we recommend the following actions to the Belmont Forum:

- Funders should make open data archiving mandatory. This has to be enforced at national levels while taking into account that the implementation of policies relies on adequate infrastructures and human support. Therefore infrastructures for data archiving have to be sufficiently supported to secure high-quality data stewardship. This in turn demands international consultations on who is archiving what kind of data which for global environmental change research could be best achieved through the Belmont Forum. This would help to prevent inefficiencies and multiplication of efforts related to public infrastructures and guidelines for data archiving.
- Scientific merits as well as accelerating research and applications are still the main motivators for publishing data; thus ethics of data sharing and re-use should be taken into account when proposing guidelines for open data sharing and re-use.
- Support and training activities should be supported in concerted ways, targeting researchers as well as current and future data and information professionals.
- Interoperability between infrastructures should be further facilitated; this can be achieved through the Belmont Forum and the Research Data Alliance. Interoperability should take into account generic requirements (e.g. providing links to publications and funder information) as well as disciplinary norms and standards (e.g. vocabularies, metadata standards).

As also pointed out by the survey there currently seems to be a lack of (awareness of) guidance; only about a quarter of the respondents was aware of guidelines for publishing data. In that sense, the Belmont Forum could be instrumental to bridge the gap between disciplines and provide a forum for the alignment and promotion of guidelines and good practices across the globe.

## Supporting Information

S1 Appendix Questionnaire of the Belmont Forum’s Open Data Survey. Questionnaire as used for the Belmont Forum’s Open Data survey; data was collected via a web survey from 16 September to 12 November 2014.
(PDF)

S2 Appendix Guidelines on Publishing Open Data. Guidelines and policies on publishing data as open data as suggested by the Belmont Forum’s Open Data survey respondents.
(PDF)
