# Supplementary material for: Open Data in Global Environmental Research: The Belmont Forum’s Open Data Survey
Source: PLoS One. 2016 Jan 15;11(1):e0146695. doi: 10.1371/journal.pone.0146695 (PMC4714918; doi:10.1371/journal.pone.0146695)
Supplement: S1 Appendix — Questionnaire as used for the Belmont Forum’s Open Data survey; data was collected via a web survey from 16 September to 12 November 2014. (PDF) [file pone.0146695.s001.pdf]

Appendix: Survey questionnaire

Questionnaire as used for the Belmont Forum's Open Data survey; data was collected via a web survey from 16 September to 12 November 2014.

All of the survey questions were non-mandatory, i.e. could be skipped by the respondents in the web survey.

Personal Information

1. Which country are you from?
2. How old are you?

Your Work

3. What is your employment role / How are you involved with data and/or climate science?

   Multiple-choice: Government, Business, Non-profit, Academia, Media, Other (please specify)

4. Which of the following are you?

   Multiple choice: Data User, Data Provider, Data Manager, Other (please specify)

5. Which discipline community do you belong to, or primarily work with, in respect to data?

   Physical sciences, Chemical sciences, Earth Sciences and environmental sciences, Biological sciences, Agricultural and veterinary sciences, Social sciences, Computer sciences, Climate and atmospheric sciences, Health sciences, Engineering, Economics, Other (please specify)

Defining and Licensing Open Data

6. Which attributes do you think are most important to open data?

   Rating scale (Likert): Very Important, Intermediate Important, Not Important

   Assessed areas: Provision of unrestricted data, Ability to re-use and re-publish data, Well defined license terms (i.e. the license is defined and it is easy to interpret the terms), Well defined quality information (i.e. data quality is defined and this information is useful for interpreting the data), Well defined metadata (i.e. metadata is complete, structured according to a standard and is useful for interpreting the data), The potential value of the data for scientific/research purposes and scholarly publishing, Easily accessible, Low cost (other than cost of publication or delivery), Free of cost, Timely, recent and actual data, Ability to protect or restrict access to some data (e.g. confidential or sensitive data), Other (please specify)

7. What access or licensing approaches do you consider most useful for open data?

   Rating scale (Likert): Very Useful, Somewhat Useful, Not Useful

   Assessed areas: Public Domain (Creative Commons CC0, Public Domain Dedication License PDDL), Attribution (Creative Commons CC-BY, Open Data Commons ODC-BY), Attribution-Share Alike (CC-BY-SA, Open Database License ODbL), Non-Commercial (CC-BY-NC), No Derivatives (CC-BY-ND), Open Government License, Other (please specify)

   For further information: `http://www.creativecommons.org/`, `http://www.opendatacommons.org/`, `https://www.nationalarchives.gov.uk/doc/open-government-licence/`.

8. Are you aware of any guidelines for publishing open data?

   Answers: Yes, No

   If your answer is 'Yes' please provide a title and/or link.

9. Do you have any concerns or comments about the licensing of data?

   Answers: Yes, No; Details

Systems and Services

10. What services/functionalities do you expect from infrastructures or systems such as networks, repositories, and visualization tools for open data when publishing or accessing data?

    Rating scale (Likert): Most Important, Intermediate Important, Not Important

    Assessed areas: The data are citable via persistent identifiers (Digital Object Identifiers (DOIs), Handles, other), Links to publications are provided, Information about tools (to process, visualize, etc.) is provided, Code can be published or accessed along with the data, Interoperability with other infrastructures or systems is supported, Publishers endorse the repository, Repositories are independently certified as "trustworthy", Usage restrictions/conditions and/or licensing information is communicated, Authorship and attribution information is highlighted, Visual interfaces to explore the data sets, Other (please specify)

Motivations and Barriers

11. In your community, how important is open data to...

    Rating scale (Likert): Very Important, Intermediate Important, Not Important

    Assessed areas: Advancing research, Educational and public outreach activities, Supporting applications of science to societal problems, Promoting capacity building and technology transfer, Other (please specify), Detail

12. Which of the following are important motivators to publish your data as open data?

    Rating scale (Likert): Very Important, Intermediate Important, Not Important

    Assessed areas: Requests from data users, Community norms, Organizational/institutional policy, Publisher policy, Funder policy, Scientific/professional society policy, Dissemination and recognition of your work/research, Acceleration of scientific research and applications, Personal commitment to open data, Other (please specify)

13. What do you see as the major barriers for publishing data as open data?

    Rating scale (Likert): Major Barrier, Barrier, Minor Barrier

    Assessed areas: Legal constraints (data privacy, national security, protection of confidentiality, intellectual property rights etc.), Organisational constraints, Commercial use and exploitation, Loss of control over intellectual property, Misinterpretation or misuse of data, Loss of credit or recognition of original work, Difficulty of clarifying data rights for work involving multiple inputs or authors, Concerns about legal liability for data or release of data, Concerns about impact of data release (e.g., on endangered species, cultural artifacts, or vulnerable populations, unwanted disclosure etc.), Desire to publish results before releasing data, Other (please specify)

Discovery and Access

14. How do you normally discover open data?

    References to data as provided in journal articles, Newsletters or other publications, Government or institutional announcements, Searching in specific data archives/repositories/systems, Web search engines, Directories or catalogs, Social media, Blogs, Direct requests to data providers, Other (please specify)

15. What specific systems or archives do you currently use to find or access open data?

    Please name up to 3 of the most important ones you would recommend and why, adding a URL where possible.

16. What specific systems or archives do you currently use to publish your data? Please name up to 3 of the most important ones you would recommend and why, adding a URL where possible.

17. Do you know of any other leading examples of open data systems either inside or outside of your community?

    Please name up to 3 of the most important ones you would recommend and why, adding a URL where possible.

18. What of the following do you consider a significant burden when accessing and reusing data for your work?

    Rating scale (Likert): Significant Burden, Some Burden, No Burden

    Assessed areas: Time delay between requesting and receiving data, Needing to register (e.g. in a website) to access data, Accessing and understanding terms of use/ licenses, Accessing usable citation/attribution information, Understanding how to access the data, Understanding how to interpret and reuse data, Varying degrees of data quality in different datasets, Varying standards in how data has been gathered, Varying data formats, Paying for data, Open data that are not timely available, Other (please specify)

19. Are there specific types, sources, or topics of data (e.g. private-sector data, household microdata, or genetic data) relevant to your work where significant expansion in open data would have substantial benefits to your work or that of your community?
