# Supplementary material for: Open Data in Global Environmental Research: The Belmont Forum’s Open Data Survey
Source: PLoS One. 2016 Jan 15;11(1):e0146695. doi: 10.1371/journal.pone.0146695 (PMC4714918; doi:10.1371/journal.pone.0146695)
Supplement: S2 Appendix — Guidelines and policies on publishing data as open data as suggested by the Belmont Forum’s Open Data survey respondents. (PDF) [file pone.0146695.s002.pdf]

Results from the Belmont Forum's Open Data Survey: Guidelines for Publishing Open Data

This document presents the references to guidelines on (open) data as provided by the respondents to the Belmont Forum's Open Data Survey.

Collected responses have been cleaned, annotated and clustered, web links to policies or guidelines have been added where possible. If not otherwise indicated the resource was mentioned once.

Licenses

- Creative Commons http://www.creativecommons.org/ 10
- Open Data Commons http://www.opendatacommons.org/ 2
- GNU General Public License, http://www.gnu.org/copyleft/gpl.html
- Open Government License, UK, https://www.nationalarchives.gov.uk/doc/open-government-licence/

Data policies by funders and organizations

- Open Data Policy Guidelines by Sunlight Foundation, http://sunlightfoundation.com/opendataguidelines/ 5
- Guidelines on Open Access to Scientific Publications and Research Data in Horizon 2020, European Commission, http://ec.europa.eu/research/participants/data/ref/h2020/grants_manual/hi/oa_pilot/h2020-hi-oa-pilot-guide_en.pdf 4
- Guidelines on Data Management in Horizon 2020, Version 1.0, 11 December 2013, European Commission, http://ec.europa.eu/research/participants/data/ref/h2020/grants_manual/hi/oa_pilot/h2020-hi-oa-data-mgt_en.pdf
- American Geosciences Union Data Policy http://publications.agu.org/author-resource-center/publication-policies/data-policy/
- OECD Science and technology policy, 2004, http://www.oecd.org/science/sci-tech/sciencetechnologyandinnovationforthe21stcenturymeetingoftheoecdcommitteeforscientificandtechnologicalpolicyatministeriallevel29-30january2004-finalcommunique.htm
- OECD Principles and Guidelines for Access to Research Data from Public Funding, 2007, http://www.oecd.org/sti/sci-tech/38500813.pdf 4
- Research Councils UK (RCUK) Policy on Open Access, http://www.rcuk.ac.uk/research/openaccess/
- Helmholtz Association policy on open access, http://www.helmholtz.de/en/research/open_access/
- German Research Foundation, Proposals for Safeguarding Good Scientific Practice, Memorandum, Wiley, Weinheim, 2013 (revised edition), http://www.dfg.de/download/pdf/dfg_im_profil/reden_stellungnahmen/download/empfehlung_wiss_praxis_1310.pdf

- G8 Open Data Charter and Technical Annex, 18 June 2013, https://www.gov.uk/government/publications/open-data-charter/g8-open-data-charter-and-technical-annex
- National Science Foundation (NSF), Responsible Conduct of Research, http://www.nsf.gov/bfa/dias/policy/rcr.jsp
- NIH Mandates guidelines, e.g. Public Access Policy, http://publicaccess.nih.gov/; Data Sharing Policy, http://grants.nih.gov/grants/policy/data_sharing/
- Natural Environment Research Council (NERC) Data Policy, http://www.nerc.ac.uk/research/sites/data/policy/
- Finnish Open Science and Research Initiative, http://openscience.fi/

Data archives/repositories and related e-infrastructures

- OpenAIRE, Open Access Infrastructure for Research in Europe (EU-funded), www.openaire.eu 1
- CGIAR Consortium of International Agricultural Research Centers, http://www.cgiar.org/consortium-news/data-standards-making-cgiar-data-available-and-accessible-2/, http://www.cgiar.org/consortium-news/cgiar-consortium-now-officially-open-access/ 2
- PANGAEA guidelines, http://www.pangaea.de/about/, http://wiki.pangaea.de/wiki/Data_submission 10
- FLuxNet Climate Data Guide http://fluxnet.ornl.gov/, https://climatedataguide.ucar.edu/climate-data/fluxnet
- European Vegetation Archive (EVA), Data Property and Governance Rules, http://euroveg.org/download/eva-rules.pdf
- Carbon Dioxide Information Analysis Center (CDIAC), http://cdiac.ornl.gov/oceans/submit.html
- CEDAR/NCAR database, https://cedarweb.vsp.ucar.edu/wiki/index.php/Data_Services:Main
- Federation of Earth Science Information Partners (ESIP Federation), Interagency Data Stewardship Principles, http://commons.esipfed.org/node/419 2
- Dryad, http://datadryad.org/themes/Mirage/docs/TermsOfService-Letter-2013.08.22.pdf, http://datadryad.org/pages/faq#depositing 6
- DataONE Best Practices, https://www.dataone.org/best-practices 3
- GBIF Data Publishing, www.gbif.org, http://www.gbif.org/publishingdata/summary, Best practice guide for Data Discovery and Publishing Strategy and Action Plans, http://www.gbif.org/resources/2614, http://www.gbif.org/publishingdata/summary, http://www.gbif.org/resources/2760, http://www.gbif.org/resources/2533 4
- Ocean Data Publication Cookbook, UNESCO 2013, www.iode.org/mg64 3
- CReATIVE-B. Services for the Biodiversity Community, Analysis Report on the legal, financial, and governance aspects in relation to the interoperability of biodiversity infrastructures and their operations, September 2012, http://creative-b.eu/documents/10826/555fc6a9-92c0-4bc4-8a3a-952a0872e996
- Ecological Archives, e.g. Instructions for Data Papers, http://esapubs.org/archive/instruct_d.htm
- SeaDataNet, Pan-European Infrastructure for Ocean & Marine Data Management, www.seadatanet.org
- Centro Italiano Studi Ornitologici, http://www.ornitho.it/
- Canadensys, e.g. 7-step guide to data publication, http://www.canadensys.net/publication/data-publication-guide

- NEEShub, Network for Earthquake Engineering Simulation, https://nees.org/
- EPILEPSIAE EU FP7 project, http://www.epilepsiae.eu/
- National Oceanic and Atmospheric Administration (NOAA), Protecting your privacy online, http://www.noaa.gov/privacy.html
- Earth System Grid Federation, http://esg-dn1.nsc.liu.se/esgf-web-fe/
- British Oceanographic Data Centre, World Ocean Circulation Experiment (WOCE), http://www.bodc.ac.uk/products/collaborative_products/woce/
- International Polar Year, http://nas-sites.org/us-ipy/, International Polar Year Data Policy, http://classic.ipy.org/Subcommittees/final_ipy_data_policy.pdf (broken link), available via Internet Archive: https://web.archive.org/web/20140806062151/http://classic.ipy.org/Subcommittees/final_ipy_data_policy.pdf 2
- NASA Moderate Resolution Imaging Spectroradiometer (MODIS), i.e. Land Processes Distributed Active Archive Center (LP DAAC), https://lpdaac.usgs.gov/dataset_discovery/modis
- CODATA, http://www.codata.org/
- Network for the Detection of Mesopause Change (NDMC) Data Policy, http://andromeda.caf.dlr.de/shared_content/ndmc/NDMC-Data_Sharing_Principles.pdf
- Atlantic Zone Monitoring Program (AZMP), http://www.meds-sdmm.dfo-mpo.gc.ca/isdm-gdsi/azmp-pmza/index-eng.html
- Drupal Ecological Information System (DEIMS), https://data.lter-europe.net/deims/
- Tropical Rainfall Measuring Mission (TRMM), http://trmm.gsfc.nasa.gov/
- Climate and Forecast (CF) Conventions and Metadata, http://cfconventions.org/
- AERONET (AErosol RObotic NETwork) data, http://aeronet.gsfc.nasa.gov/
- Berkeley Water Center, http://bwc.berkeley.edu/Amflux/fairuse.htm
- Land Processes Distributed Active Archive Center (LP DAAC), Citing our data, https://lpdaac.usgs.gov/citing_our_data
- International Council for the Exploration of the Sea (ICES) Data Policy, http://ices.dk/marine-data/Documents/ICES_Data_Policy_2012.pdf, November 2012,
- NASA Space Science Data Coordinated Archive, http://nssdc.gsfc.nasa.gov/space/
- NASA community standards, e.g. data providers get appropriate credit for their efforts. E.g.: Data Management Plan for SEAC4 RS Airborne Field Study, http://www-air.larc.nasa.gov/missions/seac4rs/docs/SEAC4RS_data_management_plan_v16.pdf
- TERENO data policy (note: data policy does not seem to be publicly available), http://teodoor.icg.kfa-juelich.de/ibg3searchportal/index.jsp
- Integrated Carbon Observation System (ICOS) Data Policy, May 2013, http://www.socat.info/upload/ICOS_data_policy.pdf
- EBAS Data Policy, Norwegian Institute for Air Research, http://ebas.nilu.no/
- NASA Planetary Data System, https://pds.nasa.gov/
- European Space Agency’s Planetary Science Archive, http://www.rssd.esa.int/index.php?project=PSA
- Network for the Detection of Atmospheric Composition Change (NDACC), http://www.ndsc.ncep.noaa.gov/data/
- Aerosol Robotic Network (AERONET), http://aeronet.gsfc.nasa.gov/
- International Council for Science – World Data System (ICSU-WDS) Data Policy, August 2012, http://www.icsu-wds.org/services/data-policy

- Group on Earth Observations – Air Quality (GEO AQ), https://www.earthobservations.org/cop.shtml
- Open Geospatial Consortium (OGC), http://www.opengeospatial.org/
- National Oceanographic Data Center (NODC), https://www.nodc.noaa.gov/
- INSPIRE, Infrastructure for Spatial Information in the European Community, http://inspire.ec.europa.eu/
- French Geological Survey, http://www.brgm.eu/
- European Nucleotide Archive, e.g. Standards and Policies, https://www.ebi.ac.uk/ena/standards-and-policies
- GenBank Submission Tools, http://www.ncbi.nlm.nih.gov/genbank/submit
- OpenARCC, data management support initiative for participants in the Adaption and Resilience in the Context of Change network (ARCC), funded by the Engineering and Physical Sciences Research Council (EPSRC), http://openarcc.wordpress.com/the-guide/
- NERC Data Citation Guidelines for Scientists, http://www.nerc.ac.uk/research/sites/data/doi/data-citation-guidelines/
- PlanetData, Network of Excellence on large-scale Data Management, EU-funded project (10/2010-09/2014), http://www.planet-data.eu/

Data & software platforms

- Dataverse, http://dataverse.org
- CKAN, Publisher Profile and Documentation, http://docs.ckan.org/en/ckan-1.8/publisher-profile.html
- Runmycode, http://www.runmycode.org – allows users to store code and data, and creates a webpage that links data, code and associated research papers
- ResearchCompendia, researchcompendia.org – allows users to create a webpage that links data, code and associated research papers
- Figshare, figshare.org
- GitHub, github.com/
- Socrata Open Data Guide, targeting organizations and government, http://www.socrata.com/open-data-guide-chapter/the-data-plan/
- Swirrl Linked Data Platform, http://www.swirrl.com/publishmydata
- LOD2 (FP7 project), collection of tools to support Linked Data publication, http://stack.lod2.eu/blog/?page_id=190

Institutional data policies and services

- University of Edinburgh, Research data management guidance, http://www.ed.ac.uk/schools-departments/information-services/services/research-support/data-library/research-data-mgmt 2
- University of Leicester, Data management support for researchers, http://www2.le.ac.uk/services/research-data
- DataUp, merged with Dash, University of California, http://dataup.cdlib.org/, https://dash.cdlib.org/
- MIT Libraries, Data management, http://libraries.mit.edu/data-management/
- Bibliothek Wissenschaftspark Albert Einstein, Open Access, http://bib.telegrafenberg.de/en/publishing/open-access/

Declarations, handbooks and general information

- Bouchout Declaration http://plazi.org/?q=bouchout 2
- Panton Principles http://pantonprinciples.org/, http://pantonprinciples.org/ 2
- Science Commons Protocol for Implementing Open Access Data http://sciencecommons.org/projects/publishing/open-access-data-protocol/
- Information Platform open-access.net http://open-access.net/de_en/homepage/
- Open Data Handbook http://opendatahandbook.org/ 3
- Guides to Publishing Data, FUMIOPEN blog post, http://fumiopen.blogspot.com/2014/09/guides-to-publishing-open-data.html
- Berlin Declaration, http://openaccess.mpg.de/
- Open Data Institute Guides, e.g. Publisher's Guide to Open Data Licensing, http://theodi.org/guides, http://theodi.org/guides/publishers-guide-open-data-licensing 2
- MIT Libraries, Data Management, http://libraries.mit.edu/data-management/
- 5 star open data http://5stardata.info/ 3
- Wikipedia http://www.wikipedia.org/
- ResearchGate http://www.researchgate.net
- Reproducibility in Science, A Guide to enhancing reproducibility in scientific results and writing, http://ropensci.github.io/reproducibility-guide/

Open Government Data Policies

- Open Government Data UK, Open Standards principles https://www.gov.uk/government/publications/open-standards-principles/open-standards-principles, http://data.gov.uk/open-data-strategies 3
- Open Government Implementation Model (Vienna), http://www.kdz.eu/en/open-government-implementation-model
- Australian Government, Open Data Toolkit, http://toolkit.data.gov.au
- South Australian Government Data Directory, http://www.data.sa.gov.au/about
- Executive Order - Making Open and Machine Readable the New Default for Government Information, The White House, Press Release, 9 May 2013, http://www.whitehouse.gov/the-press-office/2013/05/09/executive-order-making-open-and-machine-readable-new-default-government-
- Open Government Data for Citizen Engagement (OGDCE) Guidelines, 2nd Edition, United Nations, Department of Economic and Social Affairs, New York, 2013, http://www.unpan.org/DPADM/Themes/OpenGovernmentDataandServices/tabid/1536/language/en-US/Default.aspx RCUK Policy on Open Access
- Open Data France http://opendatafrance.net/documentation/
- Project Open Data, US Government, http://project-open-data.github.io/ or https://project-open-data.cio.gov/
- NZ Government guidelines https://data.govt.nz/
- The Australian Government Open Data Toolkit, http://toolkit.data.gov.au
- Open Government Data Principles, opengovdata.org
- GovData, Datenportal für Deutschland, govdata.de
- European Union Open Data Portal, open-data.europe.eu
- Civic Commons, Open Data Guidelines http://wiki.civiccommons.org/Open_Data_Guidelines
- Agency for Digital Italy, Open Data, http://www.agid.gov.it/dati-pubblici-condivisione/open-data

Data publishing and data journals

- Earth System Science Data (ESSD), data journal http://www.earth-system-science-data.net/
- Pensoft Data Publishing, http://www.pensoft.net/page.php?P=23
- Force11 Joint declaration on Data Citation Principles, https://www.force11.org/datacitation 2
- Directory of Open Access Journals (DOAJ), Open Access Information, http://doaj.org/oainfo
- Geoscience Data Journal, Wiley, http://eu.wiley.com/WileyCDA/WileyTitle/productCd-GDJ3.html 2
- Nature Publishing Group Data policies, http://www.nature.com/sdata/data-policies 2
- European Geosciences Union journal protocols (note: not quite clear what is meant), http://www.egu.eu/publications/open-access-journals/
- Springer, http://www.springer.com/
- American Geosciences Union (AGU) journal data sharing policies, http://www.slideshare.net/INSTAAR/data-citation-new-agu-guidelines 2
- PLoS guidance on Data Availability, http://journals.plos.org/plosone/s/data-availability, and recommended repositories, http://journals.plos.org/plosone/s/data-availability#loc-recommended-repositories
- Data paper guidelines, e.g. at Open Health Data, http://openhealthdata.metajnl.com/about/editorialPolicies#focusAndScope

Technical guides

- W3C Best Practices for Publishing Linked Data http://www.w3.org/TR/ld-bp/ 3
- W3C, Publishing Open Government Data, http://www.w3.org/TR/gov-data/ 5
- W3C Semantic Web Best Practices and Deployment Working Group, http://www.w3.org/2001/sw/BestPractices/
- Information and documentation -- Digital object identifier system (ISO 26324:2012), http://www.iso.org/iso/catalogue_detail?csnumber=43506

Literature

- Edmunds SC, Pollard TJ, Hole B, Basford AT. Adventures in data citation: sorghum genome data exemplifies the new gold standard. BMC Research Notes. 2012;5: 223. doi: http://dx.doi.org/10.1186/1756-0500-5-223
- Taylor KE, Stouffer RJ, Meehl GA. An overview of CMIP5 and the Experiment Design. Bulletin of the American Meteorological Society. 11/2011;93(4): 485-498. doi: 10.1175/BAMS-D-11-00094.1
- Goodman A, Pepe A, Blocker AW, Borgman CL, Cranmer K, Crosas M, et al. (2014) Ten Simple Rules for the Care and Feeding of Scientific Data. PLoS Comput Biol 10(4): e1003542. doi:10.1371/journal.pcbi.1003542
- Whitlock, MC. Data archiving in ecology and evolution: best practices. Trends in Ecology & Evolution. 2011;26(2): 61-65.
- Penev L, Mietchen D, Chavan V, Hagedorn G, Remsen D, Smith V, Shotton D. (2011). Pensoft data publishing policies and guidelines for biodiversity data. Pensoft. (29 October 2012.
- Hadley Wickham: Tidy Data. Journal of Statistical Software. 2011;59(10), Sep 2014, http://www.jstatsoft.org/v59/i10/
- Villazón-Terrazas B, Vilches-Blázquez LM, Corcho O, and Gómez-Pérez A. Methodological Guidelines for Publishing Government Linked Data. Preprint. https://www.lri.fr/~hamdi/datalift/tuto_inspire_2012/Suggestedreadings/egovld.pdf

- Boulton, G (ed.). Science as an Open Enterprise, The Royal Society, June 2012, https://royalsociety.org/topics-policy/projects/science-public-enterprise/report/
- White EP, Baldridge E, Brym ZT, Locey KJ, McGlinn DJ, Supp SR. Nine simple ways to make it easier to (re)use your data. Ideas in Ecology and Evolution 2013;6(2): 1–10. doi:10.4033/iee.2013.6b.6.f
- Sobéraná technologia, Spanish magazine, http://www.plateforme-echange.org/IMG/pdf/dossier-st-cast-2014-06-30.pdf

Other comments

- Advances in global change research require open science by individual researchers
- Many journals have guidance on these issues, as well as World Data Centres
- Many journals provide suggestions in their instructions to authors.
- Agency policies. Federal open data policies in US.
- You must follow set protocols for your country
- data journals, repositories
- Acknowledgement and citation. But very little on data limitation and caveats. Need good practices and examples of good and poor use.
- Data Management Plan Templates created to help researchers address US federal requirements for data plans.
- Of course there are guidelines, but I don't have time to look them up now -sorry!
- I just know they exist, my colleagues manage it
- I have never had large datasets that I could have published, so I never looked into it. I do use datasets for my research that I purchased or downloaded.
- You can get a doI number for a data set, which then gives the data created something tangible in terms of citations.
- Not 100 % relevant but the use of dois for publishing data
- I am also aware of DOIs and other persistent identifiers, data journals, datacite.org and several other initiatives.
- I don't understand the question.

Other, reference not identified

- ITAR – unclear reference
- Those specified by Data Science Systems - unclear reference
- forestplot.org - unclear reference, e.g. Forest Plot Database, http://erenweb.org/data/c-storage/
- Google Guide on Publishing Linked Data, https://code.google.com/p/lmf/wiki/GuidePublishingLinkedData - broken link
